# The relationship between linguistic features of speech and psychological characteristics in schizophrenia spectrum disorders

**DOI:** 10.1192/j.eurpsy.2023.1313

**Published:** 2023-07-19

**Authors:** N. D. Semenova, N. Sizova

**Affiliations:** 1 Psychological Counseling, Moscow Research Institute of Psychiatry; 2 Clinical Psychology, Pirogov Russian National Research Medical University, Moscow, Russian Federation

## Abstract

**Introduction:**

Text analysis can significantly enrich ideas about the functioning of the psyche in mental illness.

**Objectives:**

The purpose of this study was to identify the linguistic features of texts written by people with schizophrenia spectrum disorders and to study the relationship between them and the personality traits and indicators of the standard of living of patients.

**Methods:**

Twenty-nine patients with schizophrenia spectrum disorders (F20, F21, F23, F25 according to ICD-10) and 37 without mental disorders participated. All participants wrote a text on a given topic and filled in psychodiagnostic methods: a short version of the Big Five method (TIPI-RU), Q-Les-Q, and MOS SF-36. The text was analyzed using the phpMorphy morphological analysis library.

**Results:**

A comparative analysis showed that in the speech of patients, there are fewer adjectives and more verbs than in the speech of healthy subjects (p<0.05) and that the volume of speech production in patients is significantly reduced (<0.001). The results of correlations of such data with the volume of words were contradictory. A statistically significant inverse relationship was found between the verbality index and the factors of extraversion and openness to experience in the clinical group. As for the indicators of quality of life, expectedly positive correlations between the use of adjectives and negative correlations of the use of verbs with the quality of life were revealed.

**Conclusions:**

Studying the linguistic features of the speech of patients with mental disorders is essential. These indicators can be helpful in the diagnosis and treatment of the disease.

**Disclosure of Interest:**

None Declared

